# Periodontal Disease and Its Association with Metabolic Syndrome—A Comprehensive Review

**DOI:** 10.3390/ijms241613011

**Published:** 2023-08-21

**Authors:** Itay Aizenbud, Asaf Wilensky, Galit Almoznino

**Affiliations:** 1 Medical Corps, Israel Defense Forces, Jerusalem 60930, Israel; itay.aizenbud@mail.huji.ac.il; 2 Department of Periodontology, Hadassah Medical Center, Faculty of Dental Medicine, Hebrew University of Jerusalem, Jerusalem 91120, Israel; asafw@ekmd.huji.ac.il; 3 Faculty of Dental Medicine, Hebrew University of Jerusalem, Israel, Big Biomedical Data Research Laboratory, Dean’s Office, Hadassah Medical Center, Jerusalem 91120, Israel; 4 Faculty of Dental Medicine, Hebrew University of Jerusalem, Department of Oral Medicine, Sedation & Maxillofacial Imaging, Hadassah Medical Center, Jerusalem 91120, Israel

**Keywords:** periodontitis, metabolic syndrome, insulin resistance, dyslipidemia, diabetes mellitus, cardiovascular disease, obesity, osteoporosis

## Abstract

Periodontal disease is a complex and progressive chronic inflammatory condition that leads to the loss of alveolar bone and teeth. It has been associated with various systemic diseases, including diabetes mellitus and obesity, among others. Some of these conditions are part of the metabolic syndrome cluster, a group of interconnected systemic diseases that significantly raise the risk of cardiovascular diseases, diabetes mellitus, and stroke. The metabolic syndrome cluster encompasses central obesity, dyslipidemia, insulin resistance, and hypertension. In this review, our objective is to investigate the correlation between periodontal disease and the components and outcomes of the metabolic syndrome cluster. By doing so, we aim to gain insights into the fundamental mechanisms that link each systemic condition with the metabolic syndrome. This deeper understanding of the interplay between these conditions and periodontal disease can pave the way for more effective treatments that take into account the broader impact of managing periodontal disease on the comprehensive treatment of systemic diseases, and vice versa.

## 1. Introduction

Periodontitis is a chronic inflammatory disease characterized by dysbiosis and a shift in the subgingival plaque towards Gram-negative microbiota, leading to the destruction of the tooth-supporting structures in a host-mediated manner [1,2]. According to the World Health Organization (WHO), periodontitis is a significant risk factor for tooth loss. It affects approximately 40% of individuals above 30 years old in the USA and around 60% of those above 65 years old [3]. Furthermore, periodontitis has been associated with various medical conditions, including diabetes mellitus [4]. Additionally, life-threatening medical conditions such as cardiovascular diseases and metabolic syndrome have been linked to periodontitis [5,6,7].

The major parameters used to define periodontitis include bleeding on probing (BOP), gingival index (GI), plaque index (PI), periodontal pocket depth (PD), and clinical attachment loss (CAL) [1].

The metabolic syndrome, also known as “Syndrome X”, is a cluster of conditions such as central obesity, dyslipidemia, insulin resistance, and hypertension [6,8] that relate to one another, and together, they are known to increase the risk of cardiovascular diseases and type 2 diabetes mellitus (T2DM) [6]. Moreover, the syndrome was found to be associated with fatty liver [9] and obstructive sleep apnea [10]. The definition of the metabolic syndrome itself varies across different guidelines and associations. The definitions most commonly used are those written by the National Cholesterol Education Program (NCEP) Adult Treatment Panel III (ATP III) and the International Diabetes Federation (IDF). The NCEP ATP III in 2001 released guidelines for diagnosis of the metabolic syndrome [11], and they were updated in 2005 [12,13]. It defined metabolic syndrome as occurring if three out of five of the following are present: abdominal obesity, measured by waist circumference ≥102 cm (40 in) in men and ≥88 cm (35 in) in females; serum triglycerides ≥150 mg/dL (1.7 mmol/L) or drug treatment for elevated triglycerides; serum high-density lipoprotein (HDL) cholesterol <40 mg/dL (1 mmol/L) in males and <50 mg/dL (1.3 mmol/L) in females or drug treatment for low HDL cholesterol; blood pressure ≥130/85 mmHg or drug treatment for elevated blood pressure; and fasting plasma glucose (FPG) ≥100 mg/dL (5.6 mmol/L) or drug treatment for elevated blood glucose. The last update of guidelines from the International Diabetes Federation (IDF) was in 2006. The current guidelines recommend the diagnosis of an individual with metabolic syndrome if three of the following are present: increased waist circumference, normalized to different ethnic-specific waist circumference cut-points; triglycerides ≥150 mg/dL (1.7 mmol/L) or treatment for elevated triglycerides; HDL cholesterol <40 mg/dL (1.03 mmol/L) in males or <50 mg/dL (1.29 mmol/L) in females or treatment for low HDL; systolic blood pressure ≥130, diastolic blood pressure ≥85, or treatment for hypertension; and FPG ≥ 100 mg/dL (5.6 mmol/L) or previously diagnosed type 2 diabetes. In the urban population in the USA, the IDF criteria found 15–20% more individuals with metabolic syndrome compared to the NCEP ATP III criteria. Overall, in determining the absence or presence of the metabolic syndrome, the two definitions overlapped in 93% of diagnoses [14].

In recent years, metabolic syndrome has been acknowledged as a major global epidemiological concern, with a reported prevalence of 17–32% in the general population [15]. The primary connection between periodontitis and other systemic diseases associated with the metabolic syndrome cluster, like diabetes [16], cardiovascular diseases [17], and obesity [18], appears to be the inflammatory burden. This review aims to explore the associations between periodontal disease and other systemic medical conditions related to metabolic syndrome, understand how treating one affects the other, and learn about the pathological mechanisms linking them.

This review provides a comprehensive, original, and thorough perspective on the relationship between periodontitis and individual components of the metabolic syndrome as well as the metabolic syndrome as a whole.

## 2. Methods

### 2.1. Search Strategy

Data were searched online from two databases: MEDLINE and Embase. The data were searched four times on 31 December 2021; 10 August 2022; 1 March 2023; and 28 July 2023.

### 2.2. Inclusion Criteria

The search was based on the following inclusion criteria: (1) publication restricted to English, (2) studies conducted on humans, and (3) studies that were published between the years 2010–2022. Furthermore, publications with no age and sex limit were included in this review. Some relevant key publications related to the different aspects of this review were manually added even though they were published before 2010.

### 2.3. Exclusion Criteria

This review did not include publications that met the following criteria: (1) publications that were not focused on the association between periodontal disease and systemic diseases or (2) publications that did not meet these aims of the review, and (3) publications with fewer than 30 subjects.

### 2.4. Extraction of Publications from the Electronic Databases

Based on the inclusion and exclusion criteria, we used a structured search strategy defined by controlled vocabulary thesaurus terms, which are listed in Appendix A. Table 1 shows the publications extracted from the two databases based on the search strategy using these terms for each systemic disease and its association with periodontal disease.

### 2.5. Method for the Selection of Papers Extracted from the Databases

**Scheme 1:**
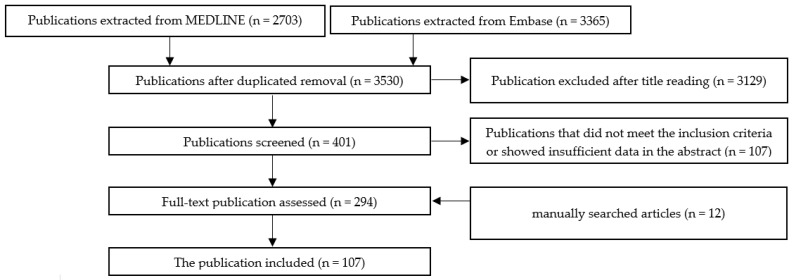
The selection method for publications included in this review.

**Scheme A1:**
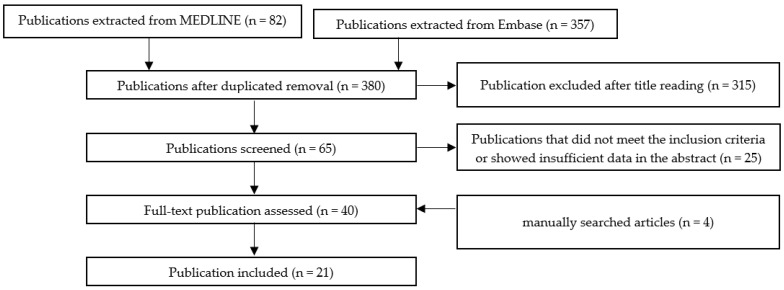
The selection method for publication assessing the association between periodontal disease and metabolic syndrome.

**Scheme A2:**
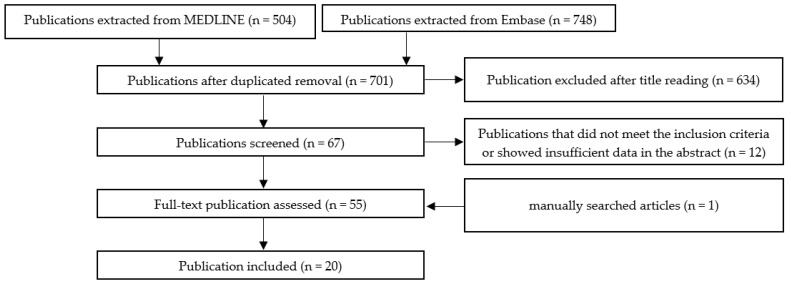
The selection method for publication assessing the association between periodontal disease and obesity.

**Scheme A3:**
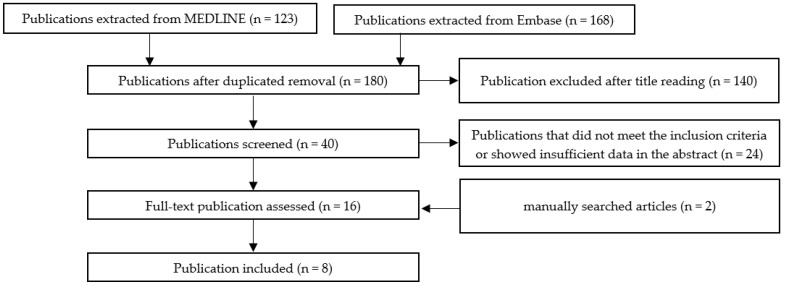
The selection method for publications assessing the association between periodontal disease and insulin resistance.

**Scheme A4:**
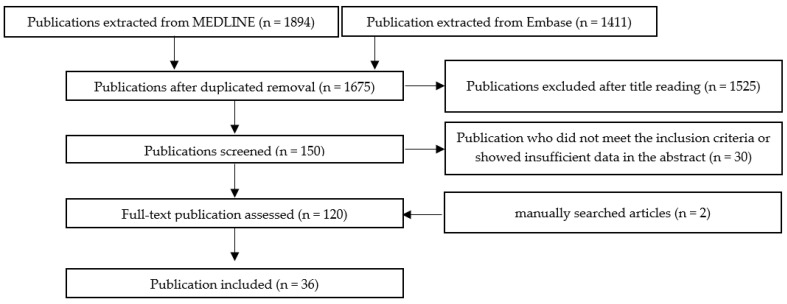
The selection method for publications assessing the association between periodontal disease and Diabetes mellitus.

**Scheme A5:**
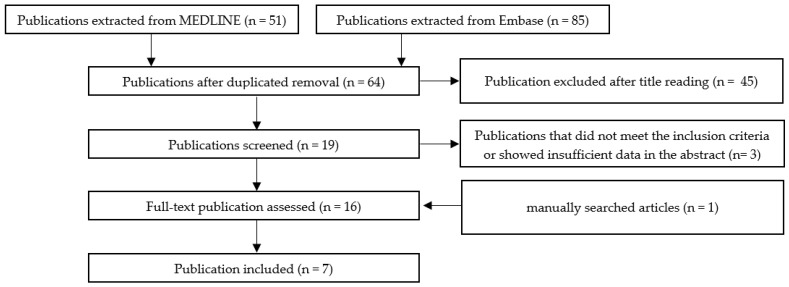
The selection method for publication assessing the association between periodontal disease and hyperlipidemia.

**Scheme A6:**
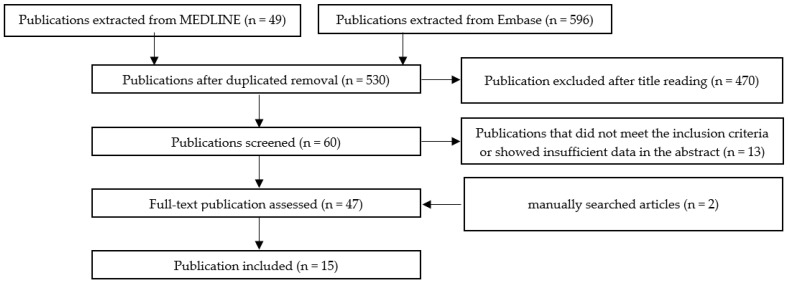
The selection method for publication assessing the association between periodontal disease and hypertension.

The method for selecting the papers extracted from the databases is shown in Scheme 1. In addition, individual schemes for each of the components of the metabolic syndrome cluster are shown in Appendix B (Scheme A1—metabolic syndrome, Scheme A2—obesity, Scheme A3—insulin resistance, Scheme A4—diabetes mellitus, Scheme A5—hyperlipidemia, and Scheme A6—hypertension).

### 2.6. Method for the Selection of Papers Extracted from the Databases

The method for selecting the papers extracted from the databases is shown in Scheme 1 and Appendix B. After the first filtering of duplicates, a second selection was made by reading the titles. After the title, the abstract was read and quality assessment of the whole article were performed. In the end, the final articles were selected for writing this review. This review was conducted using the following research engines: Embase and PubMed. This review was limited to articles published between 2010 and 2022 with manually searched-for key publications, publications in English, and studies conducted on humans. To identify the paper, the keyword and free term included the following: “insulin resistance syndrome” OR “metabolic syndrome” OR “syndrome X” OR “insulin resistance syndrome” OR “Insulin Resistance” OR “Resistance, Insulin” OR “Insulin Sensitivity” OR “Sensitivity, Insulin” OR “resistance, insulin” OR “diabetes mellitus” OR “diabetes” OR “Hyperlipidemias” OR “Hyperlipemia” OR “Hyperlipemias” OR “Hyperlipidemia” OR “Lipidemia” OR “Lipidemias” OR “Lipemia” OR “Lipemias” OR “hyperlipaemia” OR “hyperlipemia” OR “hyperlipidaemia” OR “hyperlipidaemia type ii” OR “hyperlipidaemia type iii” OR “hyperlipidaemia type v” OR “hyperlipidaemias” OR “hyperlipidemic” OR “lipaemi” OR “lipemia” OR “lipidaemia” OR “obesity” OR “Abdominal obesity” OR “Blood Pressure, High” OR “Blood Pressures, High” OR “High Blood Pressure” OR “High Blood” OR “arterial hypertension” OR “cardiovascular hypertension” OR “HTN (hypertension)” OR “hypertensive disease” OR “systemic hypertension” AND periodontitis OR “periodontal disease”.

## 3. Results

### 3.1. The Epidemiological Association between Metabolic Syndrome and Periodontal Disease

Publications related to the association between metabolic syndrome and periodontal disease are summarized in Table 2.

Metabolic syndrome includes conditions such as abdominal obesity, dyslipidemia, arterial hypertension, insulin resistance, and changes in lipid metabolism [19].

Hlushchenko et al. reported that the prevalence of periodontal disease was 1.2 times higher in people with metabolic syndrome compared to healthy individuals within the age range of 25–55 years old [20]. When looking at the opposite direction, Gomes-Filho et al. found that people with moderate to severe periodontal disease were twice as likely as people without periodontal disease to have metabolic syndrome [21]. Pham et al. investigated the association between metabolic syndrome and the severity of periodontal disease and found that 21% of the individuals with metabolic syndrome had severe periodontitis compared to only 6.8% of the healthy individuals. The BOP, GI, PI, PD, and CAL were significantly higher in individuals with metabolic syndrome compared to healthy ones [22]. Furthermore, the study revealed that the severity of the periodontal disease correlated to the number of components of the metabolic syndrome. Individuals with zero to two components of the metabolic syndrome showed better periodontal parameters than those with three components, and those with four to five components presented the worst periodontal parameters among the study groups [22]. Similar results were found in a recent meta-analysis and systematic review, as the association between periodontal disease and the metabolic syndrome was found to be in a dose–response gradient; with more components of the metabolic syndrome, the association with periodontal disease was stronger [23]. Moreover, a recent clinical trial showed that periodontal treatment improved periodontal parameters in metabolic syndrome patients, but no improvement was found in metabolic parameters such as HbA1c, waist circumference, CRP levels, and more [24].

#### 3.1.1. Underlying Mechanisms Linking Periodontal Disease to Metabolic Syndrome

##### Inflammatory Mechanisms

One of the underlying mechanisms linking periodontal disease to metabolic syndrome might be due to oxidative stress [25]. As both diseases have an inflammatory nature, pro-inflammatory cytokines originating from the gingiva infiltrate the bloodstream and increase oxidative stress. The higher oxidative stress may facilitate insulin resistance and atherosclerotic changes, and both may lead to the development of the metabolic syndrome. The connection is bidirectional, as inflammatory cytokines resulting from the metabolic syndrome components may increase the oxidative stress in the gingiva, thus Impairing the ability of the periodontium to respond to a bacterial challenge, which may result in an increased risk for periodontal disease [25].

##### Microbiological Mechanisms

Another plausible mechanism linking the two diseases might be microbial-related. It is known that obesity and type 2 diabetes can alter the oral microbiome, leading to microbial dysbiosis [26]. Subjects with periodontal disease who were obese differed dramatically from subjects with periodontal disease who were not obese in their oral microbiome composition. The obese patients had lower diversity in their oral microbiome, which may have increased the risk of developing periodontal disease [6].

### 3.2. The Epidemiological Association between Obesity and Periodontal Disease

Publications related to the association between obesity and periodontal disease are summarized in Table 3.

Obesity is one of the most recognized medical conditions of the 21st century. In 2022, the WHO published a European regional report about obesity. According to this report, obesity causes about 1.2 million deaths annually in the European region. Moreover, the report stated that overweight and obesity, especially in adolescents and children, worsened after the COVID-19 pandemic [27]. Obesity is one of the fundamental characteristics of metabolic syndrome. It is usually measured by waist circumference or body mass index (BMI) (kilogram/meter^2^). An overweight adult will be considered as one with a circumference of >90 in women or >100 in men or with a BMI of 25.0–29.9 kg/m^2^, and an obese adult will be considered as one with a circumference of >105 in women or >110 in men or with a BMI of ≥30.0 kg/m^2^ [28].

The association between obesity and periodontal disease was shown to be stronger when the BMI was higher [29]. Moreover, it was claimed that weight gain and obesity might be risk factors for developing periodontitis [30,31]. A recent meta-analysis showed that individuals who become overweight have an increased risk of 1.13 times for periodontal disease compared to healthy individuals, and obese individuals have a risk of 1.33 times for periodontal disease compared to healthy individuals [32]. A prospective study from South America revealed that women who were obese had a higher rate of CAL and an increased risk of 1.64 times for periodontal disease [33]. Moreover, individuals with higher BMI, especially above 30 kg/m^2^, showed worse periodontal parameters (CAL, BOP, PI, and PD) and a higher risk of the development of periodontitis. This was true across several years, study designs, and different races and nationalities [34]. BMI was also correlated with other components of the metabolic syndrome. As such, Fentoğlu and his group published that BMI was correlated with hyperlipidemic parameters and negatively correlated with PI, PD, BOP, and CAL [35]. Several studies suggested an association between periodontal disease and obesity, while the most prominent association was between young- and middle-aged females with high BMI and weight circumference to periodontal disease [36,37]. Another meta-analysis showed similar results, indicating that women with high BMI and high waist circumference had a higher association with periodontal disease, especially between the ages of 18 and 34. Moreover, the meta-analysis found that European individuals had a higher chance of developing periodontal disease [38].

#### 3.2.1. Mechanisms

##### Inflammation

The associated mechanism between the two might be due to the role of pro-inflammatory cytokines. Pro-inflammatory cytokine production of IL-1β, TNF-α, and IL-6 was elevated by adipocytes and macrophages in the fat tissues of obese people [39]. These cytokines produce C-reactive protein, which in turn causes an acute inflammatory state [39]. TNF-α gained particular attention since this cytokine is known to play a fundamental role in periodontal disease, as it activates osteoclasts that facilitate bone resorption and periodontitis [18].

##### Oxidative Stress

Another plausible mechanism proposed that could link the two diseases is the fact that obesity elevates the production of ROS. Elevated ROS will result in the chronic activation of inflammatory mediators in the gingiva, such as the cytokines stated above. Gingival chronic inflammation will lead to alveolar bone destruction, deeper pockets, and higher CAL [18]. Interestingly, being obese does not interfere with the outcome of nonsurgical periodontal treatment [40,41,42].

##### Biochemical Pathways

Some studies found that obesity might interfere with the differentiation of osteoblasts. Adipocytes and osteoblasts share the same embryogenic origin: the pluripotent bone marrow stem cells (BMSC) [43]. It was reported that the Wnt/β-catenin signaling pathway could drive BMSC toward osteoblasts, but this pathway is inhibited in obesity. Secreted frizzled-related protein 1, an inhibitor of Wnt/β-catenin signaling, has been reported to be increased in mild obesity but falls in morbid obesity, resulting in increased marrow adipose [44].

##### Microbiology

Another approach to the connection between the two diseases may be bacterial. Obesity is known to alter the composition of the oral microbiota. One study found higher numbers of the periodontal pathogen *Tannerella forsythia* (*T. forsythia*) in the subgingival biofilms of overweight and obese individuals than in those from individuals of normal weight [45]. Moreover, it was found that obese people had elevated proportions of *T. forsythia*, *Fusobacterium* spp., and *P. gingivalis* in their saliva, regardless of whether they had a periodontal disease or a healthy periodontium [46,47]. Another study showed that people with chronic periodontitis had higher levels of perio-pathogens such as *A. actinomycetemcomitans*, *Prevotella intermedia (P. intermedia)*, *T. forsythia*, and *Fusobacterium* spp. [46].

### 3.3. The Epidemiological Association between Insulin Resistance and Periodontal Disease

Publications related to the association between insulin resistance and periodontal disease are summarized in Table 4.

Insulin resistance is a metabolic condition that is considered a prediabetic one. The condition is defined as a reduction in insulin, targeting the responsiveness of the tissues to physiological insulin levels and insulin resistance development, which leads to higher levels of glucose in the blood [48]. Fasting plasma glucose (FPG) ≥100 mg/dL (5.6 mmol/L), diagnosis of diabetes mellitus, or drug treatment for elevated blood glucose comprise one of the defining criteria of the metabolic syndrome [11]. Insulin resistance has been linked before to other components of metabolic syndrome, such as central obesity and dyslipidemia [49]. Insulin resistance has been associated with periodontal disease. Individuals with a previous diagnosis of insulin resistance showed a significantly higher level of moderate to severe periodontal disease compared to healthy individuals [50]. Conversely, in individuals with a previous diagnosis of insulin resistance, the severity of periodontal disease was higher. The study measured insulin resistance measured by the homeostasis model assessment of insulin resistance (HOMA-IR) and showed that for every 1 mm of periodontal probing depth, the HOMA-IR score was elevated by 1.04 [51].

#### 3.3.1. Mechanisms

##### Inflammation

An underlying mechanism associated with the connection between periodontal disease and insulin resistance might be an inflammatory one [51]. Insulin resistance is considered a low-level chronic inflammatory condition. It is known that in insulin resistance, there is an increase in pro-inflammatory cytokines such as interleukin (IL)-1, IL-6, and tumor necrosis factor (TNF)-α [40,52]. Another inflammatory mediator that might be associated with the disease is reactive oxygen species (ROS) [53].

##### Microbiology

Besides an inflammatory approach, a bacterial one might link periodontal disease and insulin resistance. An in vitro study found that Porphyromonas gingivalis (P. gingivalis) stimulates insulin secretion in pancreatic cell lines [54].

The connection between insulin resistance and periodontal disease is thought to be a bidirectional one. Periodontal disease is a chronic inflammatory disease, so pro-inflammatory cytokines secreted in the gingiva enter the circulation and may worsen the existing insulin resistance and even cause it to develop into diabetes mellitus [48].

It is important to note that there is a substantial lack of quality studies on the connection between insulin resistance and periodontal disease. This might be because diabetes mellitus, which is the result of insulin resistance and one of the most linked systemic conditions to periodontal disease, gained most of the attention. Another explanation might be that it is hard to detect subjects in the prediabetic stage, and most of the population receives treatment after being diagnosed with diabetes mellitus.

### 3.4. The Epidemiological Association between Diabetes Mellitus and Periodontal Disease

Publications related to the association between diabetes mellitus and periodontal disease are summarized in Table 5.

Diabetes mellitus (DM) is a metabolic disorder classified according to the etiology [55,56] as follows:

- Type 1 (T1DM).
- Type 2 (T2DM)—T2DM is the most common one, constituting 90% of the cases [55].
- Gestational diabetes (GDM).

DM is a major predisposing factor for periodontal disease. Demmer et al. reported that people with DM are about three times more likely to have periodontal disease compared to people who do not have it [57].

**Type 1 diabetes mellitus (T1DM) and periodontitis:** While T2DM is the most common, nonetheless, a correlation has been ascertained between periodontitis and type 1 diabetes mellitus (T1DM), which is less frequently encountered. T1DM is often characterized in young, healthy people. Individuals with T1DM tend to have more dental plaque and a higher incidence of chronic gingivitis, which may develop into periodontitis at younger ages [58]. Adolescents with T1DM were found to have a prevalence of periodontal disease that is about five times higher and a rapid, more pronounced periodontal breakdown [59]. Children had a higher incidence of gingivitis when they had T1DM [60,61], and children with T1DM had four times the prevalence of periodontal disease compared to healthy individuals [61,62]. Severe periodontal disease was more frequent in the poor metabolic control group than in the good metabolic control group and the controls (26.0% vs. 20.0% vs. 5.0%) [62]. The CAL was higher in adult diabetic individuals than in healthy ones by almost twofold (4.3 mm vs. 2.3 mm, respectively) [63]. Moreover, the CAL was greater in poor glycemic control individuals compared to those with good to fair glycemic control [61]. A recent review could not determine with high certainty whether periodontal therapy helps the glycemic control in T1DM patients. A total of 9 out of 10 eligible studies showed no effect of periodontal therapy on the glycemic control, while 1 did show an improvement. The authors of the review highlighted the need for high-quality, long-term studies in this field [64].

**Type 2 diabetes mellitus (T2DM) and periodontitis:** The connection between T2DM and periodontal disease has been well established before. Several studies showed that individuals with T2DM have a higher risk of developing periodontal disease (two- to threefold) compared to healthy individuals [57,65,66,67]. Moreover, previous works showed that individuals with T2DM show a more significant periodontal loss, higher CAL, and deeper periodontal pockets [55,68,69]. DM is known to affect the body in several ways.

#### 3.4.1. Mechanisms

##### Oxidative Stress

First, DM leads to a constant hyperglycemic state. Hyperglycemia is known to worsen oxidative stress through several metabolic pathways, such as the polyol pathway [70], the hexosamine pathway [71], and the activation of protein kinase C (PKC) [72]. Second, pro-inflammatory cytokines, created mainly in adipose tissue, increase insulin resistance and are a significant cause of diabetes complications.

Moreover, the insulin resistance caused by DM is also known to elevate ROS levels, increase the number of pro-inflammatory cytokines, and reduce adiponectin. All of these changes increase the systemic inflammatory burden [48].

##### Inflammation

Another plausible mechanism linking the two diseases might be related to pro-inflammatory cytokines. Some pro-inflammatory cytokines have gained special attention, such as TNF-α, IL-1β, IL-6, and IL-18 [71]. These cytokines are known to cause an elevated full-body inflammation state, which may result in bone loss and periodontal breakdown [48,71]. One of the key factors in treating DM is glycemic control. Previous studies showed that individuals with higher glycated hemoglobin (also known as hemoglobin A1C (HbA1c)) had an increased risk of periodontal disease [73,74,75]. The mechanism associated with the two diseases is probably a multifactorial one. The inflammation burden that both diseases cause has a bidirectional relationship that worsens each disease.

The inflammatory cytokines activate metalloproteinase (MMPs) [76]. Activated MMPs with ROS will result in collagen degradation and periodontal attachment loss [55]. Moreover, diabetes is also known to increase the RANKL/OPG ratio and promote osteoclastogenesis and activation, which in turn will lead to alveolar bone loss, deeper pockets, and periodontal disease [55]. Interestingly, in other dental procedures such as extraction, similar results were found. Three months after the extraction, salivary inflammatory markers such as OPG, the OPG/RANKL ratio, and total antioxidant capacity increased in both controls and diabetic patients, while IL-18, MMP-9, and RANKL decreased in both study groups, HGF was elevated only for the control group, and TNF-α showed a marked reduction in the diabetic group. Different statistical models showed that HbA1C was correlated with these inflammatory cytokines, especially OPG and RANKL [77].

The bidirectional relationship became clear when studies found that nonsurgical periodontal treatment benefits DM patients. Periodontal treatment reduced 3–4 mmol/mol (0.3–0.4%) of glycated hemoglobin after 3–4 months, but the supporting evidence that surgical root planning (SRP) may help with the glycemic control in T2DM is of low quality [78,79]. Conversely, a systematic review from 2022 showed that after 3–4 months, there was a reduction of 0.43% (4.7 mmol/mol); after 6 months, a reduction of 0.30% (3.3 mmol/mol); and after 12 months, a reduction of 0.50% (5.4 mmol/mol) of HbA1C in diabetic patients who were diagnosed with diabetes mellitus and who went through subgingival instrumentation. Due to the new findings, the authors changed their opinion about the primary outcome of periodontal therapy and glycemic control and have now increased their level of certainty about the association [80]. It is also known that periodontal treatment might help with glycemic control in patients who are prediabetic or already diabetic [81]. Conversely, increased levels of pro-inflammatory cytokines caused by insulin resistance may reach the gingiva via systemic circulation, worsening preexisting periodontal disease [82]. Another study suggests that patients who are both diabetic and smokers who went through a complete periodontal treatment improved periodontal parameters as well as metabolic parameters such as HbA1C and fasting plasma glucose while improving inflammatory markers such as CRP. This study was conducted over a short time period (up to 6 months) [83].

##### Microbiology

Another factor that might link the diseases is a microbial one. It has been established by now that patients with DM have a reduction in the oral biological and phylogenetic diversity of the microbiome compared to normal subjects [84,85]. Moreover, similar changes were found within the subgingival microbiome. Subjects with DM had lower species diversity but higher numbers of periodontal pathogens than healthy individuals [84,86,87]. This means that while the periodontium may appear healthy, patients with DM have a higher predisposition to develop periodontal disease [86,87]. Another study, which compared healthy controls both to periodontal disease patients and to diabetic patients with periodontal disease, showed that patients with periodontal disease had higher numbers of perio-pathogens (mostly pathogenic from the red group), but patients with diabetes and periodontal disease had higher numbers of periodontal pathogens which connected with other pathogens (orange group). Although no major diversity was found within the phyla of the pathogens, the authors suggested that the connection between the periodontal pathogens is the cause for the rapid progression of periodontal disease in diabetic patients [88].

### 3.5. The Epidemiological Association between Hyperlipidemia and Periodontal Disease

Publications related to the association between hyperlipidemia and periodontal disease are summarized in Table 6.

Hyperlipidemia is defined as changes in the lipid accumulation in the blood, which mainly refers to elevated levels of triglycerides (TG), high levels of low-density lipoprotein (LDL) cholesterol, and low levels of high-density lipoprotein (HDL) cholesterol [25]. Arreguin-Cano et al. found that subjects with periodontal disease had higher TG levels in their blood, lower HDL, and worse metabolic parameters [89]. Conversely, patients with hyperlipidemia showed worse periodontal parameters including BOP, CAL, and PD [90]. Another study showed that a previous diagnosis of hyperlipidemia negatively impacted individuals’ periodontal parameters in term of BOP, CAL, PD, and periodontal index (PI). Also, the study showed that plasma HDL levels were negatively correlated with CAL, but total cholesterol, LDL, and plasma triglycerides were significantly associated with BOP, CAL, PD, and PI [35].

#### 3.5.1. Mechanisms

##### Microbiology

The mechanism linking hyperlipidemia and periodontal disease might be bacterial. Low HDL cholesterol was found to be associated with higher levels of serum antibodies against *P. gingivalis*, which is known to be a marked pathogen associated with periodontitis [91]. Moreover, higher LDL cholesterol levels were associated with higher serum antibodies against *Aggregatibacter actinomycetemcomitans* (*A.actinomycetemcomitans*), and *P. gingivalis* [92].

##### Inflammation

Another plausible explanation for the link between the diseases is an inflammatory one. In an in vivo study, mice with metabolic syndrome induced by a high-fat diet showed markedly elevated osteoclastogenesis and alveolar bone loss [93]. Furthermore, there was a marked elevation in the expression of inflammatory cytokines (IL-6, monocyte chemoattractant protein1, Receptor activator of nuclear factor kappa-Β ligand (RANKL), and macrophage colony-stimulating factor), which led to further osteoclastogenesis and alveolar bone loss [93]. The mechanism hypothesized was that systemic inflammation caused an increased biosynthesis of cholesterol, which is secondary to bacteremia, and systemic lipopolysaccharide (LPS) caused by periodontal disease. Moreover, periodontal therapy combined with antihyperlipidemic therapy showed promising results in lowering serum pro-inflammatory cytokines [94].

### 3.6. The Epidemiological Association between Hypertension and Periodontal Disease

Publications related to the association between hypertension and periodontal disease are summarized in Table 7.

Hypertension is the most common cardiovascular disease (CVD) and is defined by a systolic blood pressure higher than 140 mmHg or a diastolic blood pressure higher than 90 mmHg [95]. The WHO reported in 2014 that hypertension is responsible for 51% of deaths from stroke and 45% of overall CVD mortality in all ages and ethnic groups [96]. Hypertension has a long-term effect on the heart. As of 2015, the Global Burden of Disease Committee marked it as one of the causes of hypertensive heart failure and, ultimately, heart failure [97]. Hypertension is considered fundamental in establishing metabolic syndrome. One study showed that the prevalence of hypertension was correlated to periodontal disease severity [98]. Another cohort study showed that hypertension was positively associated with periodontal disease. Moreover, it showed that individuals in a hypertensive state were more susceptible to developing periodontal disease compared to prehypertension individuals and individuals with normal blood pressure [99]. A recent meta-analysis found that individuals with periodontal disease have a higher prevalence of hypertension [100]. Another cross-sectional study showed that the odds for arterial hypertension were higher in individuals with periodontal disease. Moreover, it showed that the odds became higher as the severity of the periodontal disease worsened [101].

#### 3.6.1. Mechanisms

##### Inflammation

The association between periodontal disease and hypertension is believed to be related to the inflammatory nature of the two diseases [5]. Oral pathogens may invade the bloodstream through the periodontal pockets and release toxins, resulting in endothelial inflammation. Endothelial inflammation is known to be the first stage in many CVDs and atherosclerosis, specifically, which may later develop into hypertension [102]. Bacteria infiltrating through the periodontal pockets and their endotoxins can draw inflammatory cells to the arterial endothelium and vascular smooth muscle, causing the initiation of the coagulation process [102].

##### Microbiology

Perio-pathogens are known to cause chronic endothelial inflammation, leading to vasospasm and potential thromboses [102]. This process may be enhanced by the inflammatory cytokines coming from the periodontium with constant low-grade inflammation [102]. The cytokines cause the endothelium to produce vasoconstrictors, promoting the aggregation and adhesion of leukocytes, possibly leading to thromboses [102]. A recent study showed that hypertensive patients had higher levels of perio-pathogens (especially *P. intermedia*, *P. gingivalis*, and *F. nucleatum*) compared to normotensive patients [103].

Only a few studies have focused on periodontal treatment and its effects on blood pressure and hypertension [104,105,106]. However, studies did show that following periodontal treatment, there was a reduction of 12.5 mmHg in systolic blood pressure and 10 mmHg in diastolic blood pressure [104]. Moreover, six months after periodontal treatment, a reduction in CRP and IL-6 levels was observed, as well as a reduction in the left ventricular mass of 12.9 g [104]. Another systematic review from the Cochrane database found that patients diagnosed with periodontal disease and CVDs (not including hypertension) experienced no beneficial effect on blood pressure after periodontal treatment [106]. Moreover, the systematic review did not find added value of intensive periodontal treatment compared to supragingival scaling only [106]. Lastly, the periodontal treatment showed promising results in patients diagnosed with periodontitis and hypertension. Periodontal treatment caused a reduction in the short term of 11.20 mmHg in systolic blood pressure and 8.40 mmHg in diastolic blood pressure in hypertensive patients with periodontal disease [106]. It should be noted that the beneficial effect of periodontal therapy had only a short-term effect, while in the long term, no effect on blood pressure was found [106]. These studies suggest an association between periodontal disease and hypertension, suggesting the consideration of periodontal treatment as part of hypertensive therapy in patients with periodontal disease [107].

## 4. Discussion

Periodontal disease affects most of the adult population worldwide [3,98]. It is considered a multifactorial disease and is associated with other systemic diseases. Most of the strong association between periodontitis and other systemic diseases is connected to the cluster known as metabolic syndrome. This cluster includes obesity, hyperlipidemia, insulin resistance, and hypertension [6]. It is important to mention that the etiology of both diseases is considered multifactorial, and several association mechanisms have been proposed between the two. The mechanism linking metabolic syndrome and periodontal disease is thought to be related to the fact that both diseases are caused by inflammatory reactions, inflammatory cytokines, and elevated systemic oxidative stress [25]. As depicted in this review, in insulin resistance, there is an increase in pro-inflammatory cytokines such as interleukin (IL)-1, IL-6, and tumor necrosis factor (TNF)-α [40,52] and ROS [53]. Furthermore, in T2DM there is an increase in TNF-α, IL-1β, IL-6, and IL-18 [71], which are known to elevate HbA1C, which increases the risk for periodontal disease [73,74,75]. Moreover, the inflammatory state is known to activate MMP, which, together with ROS, will result in periodontal breakdown [76]. Also, T2DM is known to alter the RANKL/OPG ratio, which may lead to the loss of alveolar bone [55]. An in vivo study showed that a high-fat diet will result in osteoclastogenesis, elevated levels of RANKL, and alveolar bone loss [93]. Moreover, in the study, pro-inflammatory cytokines such as IL6, MCSF, and MCP-1 will further lead to alveolar bone loss [93]. In obese individuals, elevated levels of IL-1β, TNF-α, and IL-6 were found [39]. These cytokines will produce C-reactive protein, which in turn will cause an acute inflammatory state [39]. Moreover, in obese individuals, higher ROS will lead to a higher inflammatory state and periodontal breakdown [18].

Other suggestions for the associated mechanism between the two include a bacterial one. An in vitro study found that *Porphyromonas gingivalis (P. gingivalis)* stimulates insulin secretion in pancreatic cell lines [54]. Moreover, a reduction in biological and phylogenetic diversity of the oral microbiome with higher perio-pathogens was found in T2DM patients [84,85,86,87]. In individuals with low HDL cholesterol, there were higher levels of serum antibodies against *P. gingivalis* compared to healthy individuals [91]. Moreover, higher LDL cholesterol levels were associated with higher serum antibodies against *Aggregatibacter actinomycetemcomitans* (*A.actinomycetemcomitans*) and *P. gingivalis* [92]. Similarly, one study found higher numbers of *Tannerella forsythia* (*T. forsythia*) in the subgingival biofilms of overweight and obese individuals than in those from individuals of normal weight [45]. Higher levels of perio-pathogens were found in obese people [46,47]. Also, perio-pathogens are known to cause chronic endothelial inflammation, leading to vasospasm and potential thromboses [102]. This process may be enhanced by the inflammatory cytokines coming from the periodontium, leading the endothelium to produce vasoconstrictors, promoting the aggregation and adhesion of leukocytes, and possibly leading to thromboses [102]. Taken together, the suggested mechanism accounting for the association between periodontal disease and each of the components of the metabolic syndrome cluster was similar: an increased inflammatory burden through inflammatory cytokines (mostly IL-1β, IL-6, and TNF-α) which results in systemic elevated oxidative stress and the invasion of Gram-negative bacteria, which further increase the inflammatory burden and the activation of bone-resorbing agents. The bacterial and inflammatory burden from the periodontal tissue worsens the initial phase of the components of the metabolic syndrome cluster, exacerbating each component and the cluster itself [6,20,21,24,25,26,27,28,46,51,60,61,62,63].

This review aims to give a broader view of the association between periodontal disease and the components of metabolic syndrome. Here, we reviewed the association between periodontal disease and each of the metabolic syndrome cluster components and the metabolic syndrome itself. We suggest seeing the association in a broader view, as each component affects the others and increases the risk for periodontal disease. Based on this review, we also suggest that part of the treatment for the metabolic syndrome or any of its components should include a periodontal examination, as the relationship is bidirectional. Moreover, the periodontal treatment in patients with a previous diagnosis of any of the components of the metabolic syndrome should consider looking for other systemic diseases, especially other components of the metabolic syndrome, and therefore consider the treatment possibilities while considering the possible association with the metabolic syndrome.

## 5. Conclusions

Metabolic syndrome is positively associated with periodontal disease.

The associated mechanisms between periodontal disease and metabolic syndrome components were increased pro-inflammatory mediators (including different cytokines, ROS, and elevated CRP) and constant penetration of periodontal pathogenic bacteria into the bloodstream.

Periodontal status should be checked as part of the metabolic syndrome treatment, as it significantly impacts the initiation and progression of the metabolic syndrome and its components.

Future research directions should address the gap in the literature and focus on conducting multicenter longitudinal studies in different ethnic populations to study the association of periodontal disease and its treatment with each of the metabolic syndrome components and the metabolic syndrome as a whole.

## Figures and Tables

**Table 1:** Publications extracted from electronic databases using the search strategy and thesaurus terms.

Systemic Disease	MEDLINE (PubMed)	Embase	Total
Metabolic syndrome	61	334	395
Insulin resistance	110	141	251
Diabetes mellitus	1680	2293	4273
Hyperlipidemia	51	24	75
Obesity	462	640	1002
Hypertension	457	87	544

**Table 2:** Summary of included articles exploring the association between metabolic syndrome and periodontal disease.

Authors	Methods	Study Population	Etiology	Main Outcomes
Papapanou et al.	Consensus report	MI	MI	New periodontal and peri-implantitis classification.
Chaffee et al.	Systematic review and meta-analysis	70 publications	Pro-inflammatory cytokines and oxidative stress	Bidirectional association between periodontal disease and obesity.
Paul I. Eke et al.	Retrospective, multicenter study	10,683 US adults over 30 years old	Aging	Periodontal disease and worsened periodontal parameters were correlated with aging.
Pirih et al.	Review and retrospective, single-center study	Metabolic syndrome-, cardiovascular-, and diabetes-related publication with an original study of 22 dentate recruitments	Lipids; pro-inflammatory cytokines: IL-1β, IL-6, IFN-γ, and TNF-α; and oral bacteria: *P. Gingivalis*, *Treponema denticola*, and *Ta. Forsythia and actinomycetemcomitans*	There was an association between periodontitis and metabolic syndrome, diabetes, and cardiovascular disease. Metabolic syndrome and diabetes may alter the oral microbiome.
Gobin et al.	Systematic review and meta-analysis	43 publications: 32 cross-sectional studies, 8 case-control studies, and 3 cohort studies	Metabolic syndrome components	Periodontal disease was associated with metabolic syndrome.
Watanabe et al.	Review	309 papers related to metabolic syndrome and periodontitis, of which 26 are original research papers	Aging and the number of metabolic syndrome components	Periodontal disease was associated with metabolic syndrome.
Yoneda et al.	Retrospective, single-center study	210 liver biopsies and in vivo model	*P. Gingivalis*	*P. Gingivalis* is a common perio-pathogenic that may be an additional risk factor for developing or progressing nonalcoholic fatty liver disease.
Kim et al.	Retrospective, multicenter study	3320 postmenopausal women between the ages of 40 and 79 years old	Aging	No association was found between metabolic syndrome and periodontitis in postmenopausal women.
Expert Panel on Detection, Evaluation, and Treatment of High Blood Cholesterol in Adults	Expert panel	MI	MI	Guidelines, risk factors, and evidenced-based clinical practice treatment.
Genuth et al.	Review	MI	MI	Updated guidelines.
Grundy et al.	Review	MI	MI	Updated guidelines.
Admas et al.	Comparative study	4060 individuals: adults (over 18) were randomly selected and completed an interview and biomedical examination	MI	Comparison between different guidelines and criteria of the metabolic syndrome.
Nibali et al.	Systematic review and meta-analysis	20 studies	Metabolic syndrome components	Periodontal disease was associated with metabolic syndrome.
Almoznino et al.	Longitudinal	132,529 records of patients	Metabolic syndrome components	No statistically or clinically significant association was found between hypertension with dental status. The dental inflammation score was found to have no statistical association with the C-reactive protein.
Hlushchenko et al.	Retrospective, single-center study	190 individuals with metabolic syndrome and 90 healthy individuals between the ages of 25 and 55	Metabolic syndrome components	Metabolic syndrome was found to be a risk factor for periodontal disease. Periodontal disease was more prevalent and intense in individuals with metabolic syndrome. The progression of periodontal disease was faster in metabolic syndrome patients. Periodontal disease was found to be 1.2 times higher in people with metabolic syndrome compared to healthy individuals.
Gomes-Filho et al.	Case-control study	870 individuals: 408 with first metabolic syndrome diagnosis and 462 healthy individuals	Metabolic syndrome components	A positive association was found between periodontal disease and the metabolic syndrome. Periodontal disease found to be 2 times higher in people with metabolic syndrome compared to healthy individuals.
Pham et al.	Retrospective, single-center study	412 individuals, average age 57.8 ± 5.7 years: 206 patients with metabolic syndrome and 206 healthy participants	Metabolic syndrome components	Periodontal parameters were higher in metabolic syndrome patients. Individuals with metabolic syndrome had a 21% chance of developing periodontal disease compared to 6.8% in healthy individuals. The severity of the periodontal disease correlated to the number of components of the metabolic syndrome.
Campos et al.	Systematic review and meta-analysis	52 studies totaling 140,434 participants	Metabolic syndrome components	Metabolic syndrome was associated with periodontal disease in a dose–response gradient way. The higher the number of metabolic syndrome components, the stronger the association to periodontal disease.
Milanesi et al.	Randomized clinical trial	158 patients with moderate to severe periodontitis diagnosed with metabolic syndrome	Metabolic syndrome components	Periodontal treatment improved periodontal parameters but not metabolic syndrome parameters.
Lamster et al.	Review	Epidemiological, in vivo, and in vitro publication	Pro-inflammatory cytokines: TNF-α, Il-6, IL-1β, IL-17; and oxidative stress	The association between periodontal disease and metabolic syndrome was pro-inflammatory, mainly oxidative stress. Individuals with metabolic syndrome and individuals with periodontitis showed higher levels of circulating inflammatory mediators.
Tam et al.	Prospective cohort study	69 patients between 18 and 80 years of age with type 2 diabetes mellitus, obesity, and at least 10 teeth and/or implants, wisdom teeth excluded	Oral bacteria	A significant difference was found in the oral microbial composition between obese and nonobese individuals.

MI—missing information.

**Table 3:** Summary of included articles exploring the association between obesity and periodontal disease.

Authors	Methods	Study Population	Etiology	Main Outcomes
Khosravi et al.	Review	Epidemiological, in vivo, and in vitro publication	IL-6 and TNF-α	Obese individuals have higher levels of IL-6 and TNF-α, which make them more susceptible to developing destructive periodontal disease.
WHO	WHO regional report	MI	MI	The report reviews obesity and its consequences in the European region in terms of finances, cause of death comorbidities with other diseases, and the additional risk of COVID-19. Moreover, the report highlights the importance of prevention.
Ross et al.	Review	Epidemiological and retrospective publication	MI	Consensus statement that clarifies how to measure waist circumference and BMI properly in patients.
Pataro et al.	Retrospective, single-center study	594 women divided into groups based on their weight	Obesity	Periodontitis was positively correlated with weight gain and was more evident in the obesity group.
Ekuni et al.	Cohort prospective study	224 students	High BMI	An increase in BMI was associated with worsening periodontal status.
Gorman et al.	Longitudinal study	1038 men	High BMI	Obesity was positively correlated with the progression of periodontal disease.
Nascimento et al.	Systematic review and meta-analysis	Five publications were included, with 42,198 subjects enrolled	Overweight and obesity	A positive correlation was found between weight gain and new cases of periodontal disease.
Gaio et al.	Prospective study	582 individuals: 333 males and 249 females with a median age of 36.02 ± 14.97 years	Overweight and obesity	Obesity is a risk factor for clinical attachment loss, and it is more pronounced in women.
Saito et al.	Review	MI	Obesity	Obesity was negatively correlated with periodontal parameters and risk for periodontal disease.
Abu-shawish et al.	Systematic review	15 studies with a total of 6603 patients	Obesity	Association between periodontal disease and obesity was higher in women.
Carneiro et al.	Cross-sectional study	345 patients	Obesity	Association between periodontal disease and obesity was higher in women with high BMI and high waist circumference between the ages 18 and 49.
Kim et al.	Systematic review and meta-analysis	37 manuscripts	Obesity	Women in the age range 18–34 with high BMI and waist circumference had higher chances of developing periodontal disease.
Martinez-Herrera et al.	Systematic review	28 publications	Overweight and obesity	Obesity was positively correlated with the progression of periodontal disease. Obesity and weight gain recognized as risk factors for periodontitis. Pro-inflammatory cytokines are recognized as a possible link between obesity and periodontal disease.
Duzagac et al.	Retrospective, single-center study	45 patients	Obesity	Patients with periodontitis and obesity responded to periodontal therapy as well as patients with periodontitis without obesity.
Balli et al.	Retrospective, single-center study	80 patients	Obesity	Chemerin and IL-6 were positively correlated with obesity.
Pierce et al.	Review	In vivo and in vitro publications	MI	Adipocytes and osteoblasts have the same embryonic origin.
Lagathu et al.	In vivo and in vitro study	In vivo and in vitro models	Wnt/β-catenin signaling pathway	Secreted frizzled-related protein 1, an inhibitor of Wnt/β-catenin signaling, peaked in mild obesity but fell in morbid obesity, which resulted in adipose changes and metabolic changes.
Al-Rawi et al.	Retrospective, single-center study	78 saliva samples from obese and nonobese patients	Obesity	Oral perio-pathogenic bacteria such as *Fusobacterium* spp., *P. Gingivalis*, and T. Forsythia are higher in obese individuals.
Maciel et al.	Retrospective, single-center study	166 patients divided based on their weight and periodontal status	Obesity	Obesity was correlated with an increase in perio-pathogens.
De Andrade et al.	Retrospective, single-center study	67 patients divided based on their weight	Overweight and obesity	Obesity was correlated with an increase in perio-pathogens.

MI—missing information.

**Table 4:** Summary of included articles exploring the association between insulin resistance and periodontal disease.

Authors	Methods	Study Population	Etiology	Main Outcomes
Gurav et al.	Review	Epidemiological, in vivo, and in vitro publication	Pro-inflammatory mediators: TNF-α, Il-6, prostaglandins E2, and oxidative stress	Pro-inflammatory cytokines are associated with the connection between periodontal disease and insulin resistance.
Roberts et al.	Review	Epidemiological publication	Metabolic syndrome components	Insulin resistance is a significant component in the metabolic syndrome cluster. Insulin resistance and diabetes may improve by doing aerobic activity.
Benguigui et al.	Cross-sectional study	1625 subjects between the ages 35 and 74	Metabolic syndrome components	Insulin resistance is associated with periodontal disease and especially with moderate and severe periodontitis.
Demmer et al.	Retrospective, multicenter study	3616 participants, mean age (±SD) 43 ± 17 years, who received a periodontal examination and fasting blood draw	C-reactive protein, insulin resistance diagnosis	Periodontal infection was associated with insulin resistance.
Altay et al.	Retrospective, single-center study	22 dyslipidemic patients with obesity and 24 healthy individuals without obesity with generalized chronic periodontitis	Pro-inflammatory mediators: TNF-α, IL-6, and CRP	Periodontal treatment lowers the levels of circulating pro-inflammatory cytokines and may be associated with a decrease in insulin resistance in obese patients.
Abou-Raya et al.	Retrospective, single-center study	185 patients: 96 women and 89 men, mean age of 53.5 (7.3)	Pro-inflammatory mediators: TNF-α, IL-6, CRP, and ICAM-1	Periodontal therapy appears to have an impact on diabetic and prediabetic patients. Periodontal therapy reduced systemic inflammation and improved glycemic control in T2DM patients.
Marchetti et al.	Review	Epidemiological, in vivo, and in vitro publication	Pro-inflammatory mediators and oral bacteria	Oxidative stress is associated with the connection between periodontal disease and components of the metabolic syndrome.
Bhat et al.	Retrospective, single-center study	Pancreatic β cell line MIN6	*P. Gingivalis*	*P. Gingivalis* stimulates insulin secretion by the pancreatic cell line.

**Table 5:** Summary of included articles exploring the association between diabetes mellitus and periodontal disease.

Authors	Methods	Study Population	Etiology	Main Outcomes
Winning L. et al.	Prospective cohort study	1331 Irish men between the ages of 58 and 72	Periodontal disease	Men who had periodontal disease had higher chances of developing T2DM.
Duarte et al.	Retrospective, single-center study	Gingival biopsies from healthy patients with chronic periodontal disease	Pro-inflammatory mediators: TLR-2, TLR-4, IL-17, RANKL, and receptor of advanced glycation end products (RAGE)	Periodontal disease increases the expression of pro-inflammatory mediators.
Negrato et al.	Review	Epidemiological, in vivo, and in vitro publication	Diabetic parameters: HbA1c and fasting plasma glucose	Diabetes harms periodontal health and vice versa.
Mauri-Obradors et al.	Systematic review	19 studies with a total of 3712 patients	Previous diagnosis of DM	Awareness of oral manifestations of DM in both general practitioners and dentists is inadequate.
Ziukaite et al.	Systematic review and meta-analysis	27 studies	Diabetic parameters: HbA1c and fasting plasma glucose	Diabetes diagnosis is higher within periodontitis populations compared to healthy individuals.
Roy et al.	Cohort	50 persons with type 1 diabetes: 30 males and 20 females; mean age: 35.2 years	Type 1 diabetes	Patients with T1DM presented poorer oral health, more plaque, and more gingival inflammation than healthy controls, especially in the younger subjects.
Rapone et al.	Meta-analysis	10 studies	T1DM in adolescence and childhood	The association between periodontitis and T1DM is less solid than between periodontitis and T2DM.
Novotna et al.	Review	Epidemiological, in vivo, and in vitro publication	T1DM in adolescence and childhood	Younger individuals with T1DM tend to have higher amounts of dental plaque and an increased incidence of chronic gingivitis.
Graves et al.	Review	Epidemiological, in vivo, and in vitro publication	Pro-inflammatory mediators: TNF-α, IL-1, IL-6, receptor of advanced glycation end products (RAGE), and RANKL	Individuals with diabetes mellitus tend to have more periodontal disease. RANKL could be a critical factor in the association between periodontal disease and diabetes mellitus.
Popławska-Kita et al.	Retrospective, single-center study	107 patients with diabetes and 40 controls	T1DM and pro-inflammatory mediators: TNF-α, IL-1, and CRP	T1DM increases the chance of periodontal disease. The etiology is probably an inflammatory one.
Meenawat et al.	Retrospective, single-center study	28 patients diagnosed with type 1 diabetes mellitus and 20 healthy controls	T1DM	The prevalence of periodontal disease was higher in type 1 diabetes mellitus.
Reddy et al.	Review	3 systematic reviews and 9 original research papers	T1DM	Periodontal therapy did not affect the glycemic control in T1DM patients.
Leite et al.	Review	Epidemiological and retrospective publication	T2DM	The leading association between periodontal disease and diabetes mellitus is inflammation. There is a lack of knowledge among patients and dentists regarding the manifestations of diabetes mellitus in the oral cavity.
Liccardo et al.	Review	Epidemiological and retrospective publication	Pro-inflammatory mediators: TNF-α, interleukins, prostaglandins, MMP, ROS, and oral bacteria	Periodontal disease is associated with diabetes mellitus and cardiovascular diseases.
Preshaw et al.	Review	Epidemiological and retrospective publication	Pro-inflammatory mediators: TNF-α, interleukins, prostaglandins, MMP, ROS, and oral bacteria	A bidirectional relationship exists between periodontal disease and diabetes mellitus, and the main link seems to be an upregulated inflammation arising from each condition having adverse effects on the other.
Preshaw et al.	Review	Epidemiological and retrospective publication	Pro-inflammatory mediators: TNF-α, interleukins, prostaglandins, MMP, ROS, and oral bacteria	Diabetes mellitus is a significant risk factor for periodontitis, and the risk of periodontitis is greater if glycemic control is poor.
Takeda et al.	Cross-sectional	71 Japanese patients diagnosed with type 2 diabetes mellitus	Metabolic syndrome components and especially T2DM	Periodontal inflammation was associated with fasting plasma glucose and HbA1c.
Giacco et al.	Review	Epidemiological, in vivo and in vitro publication	ROS	Diabetes cause ROS overproduction in endothelial cells of both large and small vessels and the myocardium. This causes the activation of significant pathways involved in the pathogenesis of complications, one of them being the polyol pathway.
Wu et al.	Review	Epidemiological, in vivo, and in vitro publication	Pro-inflammatory mediators: TNF-α, interleukin (IL)-1β, IL-6 and IL-18, RAGE, ROS, and oral bacteria: *Porphyromonas gingivalis*, *Tannerella forsythia*, and *Treponema denticola*	Hyperglycemia hyperactivates several molecular pathways, including the polyol pathway and the hexosamine pathway. Diabetes mellitus and periodontal disease are mainly linked by the pro-inflammatory state, which is caused by pro-inflammatory mediators and perio-pathogenic bacteria.
Yan et al.	Review	Epidemiological, in vivo, and in vitro publication	Pro-inflammatory mediators: PKC, ROS, and oral bacteria	ROS-activating protein kinase C (PKC) further increases the pro-inflammatory state.
Hsu et al.	Randomized control trial	64 patients with T2DM	T2DM	Periodontal treatment improved short-term periodontal parameters and quality of life in T2DM patients.
Sundar et al.	Retrospective, single-center study	266 T2DM patients: 91 females and175 males with a mean age of 47.65 ± 5.93 years	T2DM	Periodontal treatment is associated with a reduction in HbA1c among T2DM patients.
Altamash et al.	Longitudinal study	129 individuals	T2DM	Periodontal treatment in all groups improved periodontal inflammation with a reduction in HbA1c levels at three and six months.
Arreguin-Cano et al.	Retrospective, single-center study	97 T2DM patients	T2DM	Salivary MMP-2 activity and HbA1c were positively correlated with the severity of periodontitis.
Simpson et al.	Review	35 studies, which included 2565 participants in total	T2DM	There is low-quality evidence that periodontal treatment by SRP improves glycemic control in people with diabetes.Patients with periodontal disease had additional risk factors such as low HDL cholesterol, high triglycerides, and LDL cholesterol.
Maftei et al.	In vivo study	45 healthy individuals and 41 diabetic individuals	T2DM	Dental extraction in diabetic patients increased serum pro-inflammatory markers and correlated with higher HbA1c.
Madianos et al.	Systematic review and meta-analysis	34 studies	T2DM and T1DM	Periodontal treatment improved glycemic control in diabetic patients.
Simpson et al.	Review	35 studies, which included 3249 randomized participants	T2DM	Due to new findings, the levels of certainty increased between subgingival instrumentation and reduction in HbA1C.
Chandni et al.	Retrospective, single-center study	40 diabetic patients	Diabetic parameters	Periodontal therapy can improve the periodontal status, reduce insulin resistance, and improve insulin sensitivity.
Papageorgiou et al.	Systematic review and meta-analysis	15 studies, including 867 patients	TNF-α	Overweight prediabetic individuals showed elevated inflammatory and metabolic parameter levels compared to nonobese, normoglycemic individuals.
Pham et al.	Randomized controlled trial	40 diabetic smoking patients	T2DM and smoking	Periodontal therapy improved periodontal parameters, diabetic parameters, and inflammatory parameters.
Yang et al.	Retrospective, single-center study	32 nondiabetic individuals and 70 diabetic individuals, all diagnosed with periodontitis	T2DM	Diabetic patients showed a reduction in oral bacterial diversity.
Saeb et al.	Case-control study	15 type 2 diabetes patients, 10 impaired glucose tolerance (IGT) subjects, and 19 control subjects	T2DM and IGT	Diabetic patients showed a reduction in oral bacterial diversity.
Shi et al.	Longitudinal study	31 individuals who were divided into diabetic or nondiabetic groups, and each group was subdivided into healthy or having periodontal disease	T2DM	T2DM patients are more susceptible to subgingival shifts in the microbiome toward dysbiosis, potentially due to impaired host metabolic and immune regulation.
Ganesan et al.	Retrospective, single-center study	175 nonsmoking normal glycemic individuals (controls), smokers, diabetics, and diabetic smokers with periodontitis as well as periodontally healthy controls, smokers, and diabetics	Smoking and T2DM	Diabetic patients showed a reduction in oral bacterial diversity and higher chances of shifting toward dysbiosis in the subgingival microbiota.
Yu et al.	In vitro study	Gingival samples from 18 healthy individuals, 18 patients with periodontal disease, and 11 diabetic individuals with periodontal disease	T2DM	Patients with diabetes and periodontal disease had more connections between different perio-pathogens compared to patients with periodontal disease only.

**Table 6:** Summary of included articles exploring the association between hyperlipidemia and periodontal disease.

Authors	Methods	Study Population	Etiology	Main Outcomes
Tu et al.	Comparative study	33,740 records: 18,469 women and 15,271 men	Metabolic syndrome components	An association was found between metabolic syndrome and periodontal disease. Women with periodontitis had higher triglycerides in their blood and lower HDL.
Shivakumar et al.	Comparative study	60 subjects with hyperlipidemia and 60 norm-lipidemic subjects	Hyperlipidemia	Hyperlipidemic patients had higher values of periodontal parameters compared to the control group.
Fentoğlu et al.	Retrospective, single-center study	98 patients	Hyperlipidemia	Periodontal parameters are positively correlated with hyperlipidemic parameters.
Iwaski et al.	Retrospective, single-center study	216 participants	Metabolic syndrome components	An association was found between metabolic syndrome and periodontal disease. Low HDL cholesterol was associated with higher serum antibodies to *P. gingivalis.*
Jaramillo et al.	Retrospective, single-center study	192 advanced and 256 moderate periodontitis patients compared to 229 control patients	Hyperlipidemia	Periodontal disease changes the serum lipid levels, which in turn changes the number of serum antibodies against perio-pathogens.
Li et al.	In vivo study	Mice with high-fat diet	Metabolic syndrome components and saturated fatty acids	Metabolic syndrome was associated with increased periodontal inflammation and alveolar bone loss in an animal model. Saturated fatty acids play an important role in metabolic syndrome periodontitis by enhancing the expression of LPS-induced inflammatory cytokines in macrophages.
Fentoğlu et al.	Retrospective, single-center study	52 hyperlipidemic patients and 28 healthy controls with periodontitis	Hyperlipidemia	Periodontal therapy combined with antilipidemic treatment could reduce the inflammatory state of hyperlipidemia.

**Table 7:** Summary of included articles exploring the association between hypertension and periodontal disease.

Authors	Methods	Study Population	Etiology	Main Outcomes
Tonetti et al.	Consensus report	MI	MI	Periodontitis increases the risk of future cardiovascular disease.
Paul et al.	Review	Epidemiological, in vivo, and in vitro publication	Pro-inflammatory mediators: IL-6, IL-17, TNF-α, IL-1β, and reactive oxygen species	Inflammatory agents and oxidative stress play a crucial role in the progression of both periodontal disease and cardiovascular diseases.
Williams et al.	Guidelines paper	MI	MI	New guidelines to help medical professionals manage individuals patients’ condition.
Mancia et al.	Guidelines paper	MI	MI	Updated guidelines to help medical professionals manage individuals patients’ condition.
Roth et al.	Retrospective, multicenter study	Epidemiological publication	CVD	CVD remains a major cause of death all around the globe. Areas with higher sociodemographic indexes showed dramatically decreased morbidity from CVD, but all other regions had slight or no improvement.
Xavier et al.	Retrospective observational study	260 patients who self-reported hypertension and were diagnosed with periodontal disease	Periodontal disease	Statistical difference in the prevalence of self-reported hypertension was correlated with chronic periodontitis severity.
Kawabata et al.	Prospective cohort study	2588 students (1278 males, 1310 females) aged 18.2 ± 0.7 years	Hypertension and BMI	Hypertension was positively associated with periodontal disease.
Aguilera et al.	Systematic review and meta-analysis	40 eligible studies	Hypertension	Hypertension was positively associated with periodontal disease.
Konnecke et al.	Cross-sectional study	5735 individuals	Arterial hypertension	Arterial hypertension was associated with periodontal disease. And the association was stronger as periodontal disease worsened.
Paizan et al.	Review	Epidemiological publication	Pro-inflammatory mediators, reactive oxygen species, and oral bacteria	Several pathological mechanisms link periodontal disease with CVD, especially hypertension; they include inflammation, oral pathogens, and increased oxidative stress.
Da Silveria et al.	Cross-sectional study	38 patients (14 normotensive and 24 hypertensive)	Hypertension	Hypertensive patients had higher levels of perio-pathogens.
Vidal et al.	Retrospective, single-center study	162 periodontal patients	Pro-inflammatory cytokines: CRP, IL-6, and fibrinogen	Periodontal treatment significantly reduced the level of pro-inflammatory cytokines, left ventricular mass, and arterial stiffness.
Sanz et al.	Review and consensus report	Epidemiological publication	Hypertension	The association between periodontal disease and hypertension. The possible beneficial outcome of a reduction in blood pressure after periodontal treatment.
Luo et al.	Review	8 randomized controlled trials	Hypertension	Periodontal treatments had no long-term effect on blood pressure. Suggested evidence showed a short-term effect of periodontal treatment on blood pressure reduction.
Hada et al.	Randomized controlled trial	55 patients	Hypertension and high cholesterol	Periodontal treatments lowered blood pressure and showed beneficial outcomes on the lipid profile in patients undergoing nonsurgical periodontal treatment.

MI—missing information.

**Figure A1:**
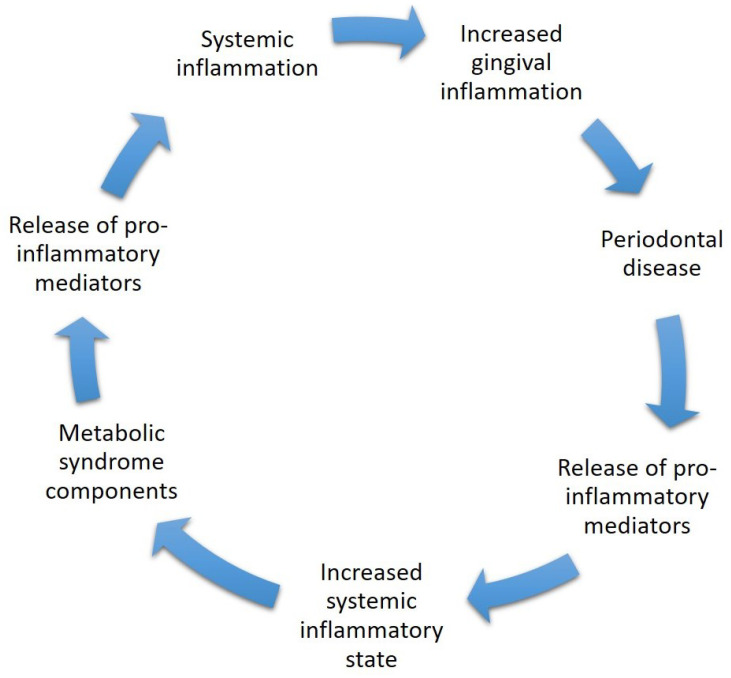
The mechanism of inflammation, including systemic inflammation, which in turn may lead to gingival inflammation and later on to periodontal disease. The gingival inflammatory state releases pro-inflammatory mediators (mainly cytokines and ROS) to the general bloodstream, which later on may cause a systemic inflammatory state which may initiate or worsen already established components of the metabolic syndrome. The components are known to increase the inflammatory state of the body by releasing pro-inflammatory mediators which in turn may cause gingival inflammation.

**Figure A2:**
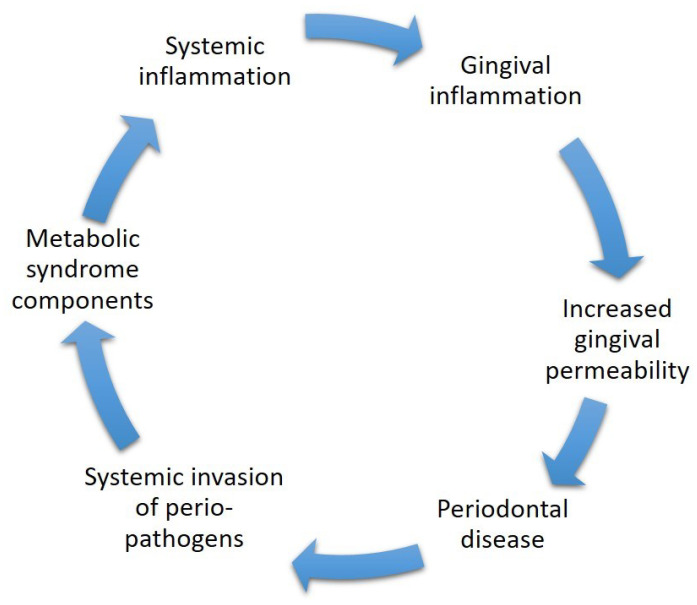
The mechanism of microbiology includes systemic inflammation caused by metabolic syndrome components which in turn may lead to gingival inflammation. The gingival inflammation causes the gingiva to become more permeable, which may later end in periodontal disease. In periodontal disease, there is an increase in perio-pathogens which may invade systemically through the bloodstream, which may initiate or worsen already established components of the metabolic syndrome. The components are known to increase the inflammatory state of the body by releasing pro-inflammatory mediators which in turn may cause gingival inflammation.

## Data Availability

No new data were created or analyzed in this study. Data sharing is not applicable to this article.

## Appendix A. Data Search Strategy

**MEDLINE (PubMed)**

#1: “periodontitis” OR “periodontal disease”.

#2: “insulin resistance syndrome” OR “metabolic syndrome” OR “syndrome X” OR “insulin resistance syndrome”.

#3: “Insulin Resistance” OR “Resistance, Insulin” OR “Insulin Sensitivity” OR “Sensitivity, Insulin”.

#4: “diabetes mellitus” OR “diabetes”.

#5: “Hyperlipidemias” OR “Hyperlipemia” OR “Hyperlipemias” OR “Hyperlipidemia” OR “Lipidemia” OR “Lipidemias” OR “Lipemia” OR “Lipemias”.

#6: “obesity” OR “Abdominal obesity”.

#7: “Blood Pressure, High” OR “Blood Pressures, High” OR “High Blood Pressure” OR “High Blood Pressures” OR “hypertension”.

#1 AND #2; #1 AND #3; #1 AND #4; #1 AND #5; #1 AND #6; #1 AND #7.

**EMBASE**

#1: “periodontitis” OR “periodontal disease”.

#2: “insulin resistance syndrome” OR “metabolic syndrome” OR “syndrome X”.

#3: “Insulin Resistance” OR “resistance, insulin”.

#4: “diabetes mellitus” OR “diabetes”.

#5: “hyperlipaemia” OR “hyperlipemia” OR “hyperlipidaemia” OR “hyperlipidaemia type ii” OR “hyperlipidaemia type iii” OR “hyperlipidaemia type v” OR “hyperlipidaemias” OR “hyperlipidemic” OR “lipaemia” OR “lipemia” OR “lipidaemia”.

#6: “obesity” OR “Abdominal obesity”.

#7: “arterial hypertension” OR “blood pressure, high” OR “cardiovascular hypertension” OR “high blood pressure” OR “HTN (hypertension)” OR “hypertensive disease” OR “systemic hypertension”.

#1 AND #2; #1 AND #3; #1 AND #4; #1 AND #5; #1 AND #6; #1 AND #7.

## Appendix B. The Selection Method for Publications

Scheme A1, Scheme A2, Scheme A3, Scheme A4, Scheme A5 and Scheme A6 show the selection methods for publications for each systemic condition analyzed with periodontitis: metabolic syndrome (Scheme A1), obesity (Scheme A2) insulin resistance (Scheme A3), diabetes mellitus (Scheme A4), hyperlipidemia (Scheme A5), and hypertension (Scheme A6). After the first filtering of duplicates, a second selection was conducted by reading the titles. After the title, the abstract was read, and quality assessment of the whole article was performed. In the end, the final articles were selected for writing this review.
